# Perioperative Spinal Cord Stimulation Combined With Targeted Muscle Reinnervation for Chronic Neuropathic Pain: A Case Report

**DOI:** 10.7759/cureus.116867

**Published:** 2026-09-24

**Authors:** Tolga Suvar, Angie Jung, Robert J McCarthy, David Kurlander

**Affiliations:** 1 Department of Anesthesiology, Rush University Medical Center, Chicago, USA; 2 Department of Plastic Surgery, Rush University Medical Center, Chicago, USA

**Keywords:** amputation, case report, neuromodulation, phantom limb pain, regenerative peripheral nerve interface, spinal cord stimulation, targeted muscle reinnervation

## Abstract

Phantom limb pain (PLP) and residual limb pain (RLP) are often difficult to treat with pharmacologic regimen alone and affect many lower extremity amputation patients. We report the case of a 56-year-old female patient with chronic PLP and a right above-the-knee amputation who underwent perioperative spinal cord stimulation (SCS) in conjunction with targeted nerve reinnervation. Following SCS implantation and surgical intervention, the patient reported sustained significant pain reduction of approximately 80% over three months, improved function, and cessation of analgesic use. This case illustrates the feasibility of sequential central neuromodulation and peripheral nerve reconstruction in refractory post-amputation pain and generates the hypothesis that these approaches may have complementary roles.

## Introduction

In the United States, approximately 150,000 patients every year undergo lower extremity amputation, mainly due to medical conditions such as diabetes mellitus, trauma, or peripheral vascular disease [1]. Among these cases, nearly 95% of patients experience amputation-related pain, with 79.9% reporting phantom limb pain (PLP) and 67.7% reporting residual limb pain (RLP) [2]. Pain in the amputated limb is frequently linked to neuroma formation at the severed nerve site. Given the limited efficacy of pharmacologic therapies, there is growing interest in neuromodulation for post-amputation pain management [3]. While peripheral nerve stimulation has shown benefit in previous studies, [4] evidence supporting the use of central stimulators such as spinal cord stimulation (SCS) remains limited [4,5].

Surgical techniques such as targeted muscle reinnervation (TMR) and regenerative peripheral nerve interface (RPNI) have been shown to reduce the incidence of neuroma-related PLP, with reported rates ranging from 0% to 55%, compared to historical rates of 70-80%, yet complete or lasting relief is uncommon [6]. We present a case of refractory post-amputation PLP managed sequentially with SCS followed two weeks later by TMR.

This case report was determined to be exempt from the Institutional Review Board review at Rush University Medical Center. Authors received a non-human subject research (IRB-118) approval for publication of this case report. The participant signed a Case Report Authorization Form, providing the investigators Health Insurance Portability and Accountability (HIPAA) authorization for access of the information in the medical record for use in this case. This report adheres to the applicable Enhancing the Quality and Transparency of Health Research (EQUATOR) guideline for Case Reports (CARE).

## Case presentation

A 56-year-old female patient with a past medical history significant for right above-the-knee amputation (AKA), chronic right PLP, seizures, deep vein thrombosis (DVT), and complex regional pain syndrome type II presented to the Rush Pain Clinic for management of intractable pain. Her limb pain began after a traumatic fall in 2019 that resulted in a femur fracture and subsequent infection. She underwent a right AKA in November 2020 due to her history of DVT and recurrent osteomyelitis. She was taking pregabalin 50 mg thrice a day, tramadol 50 mg every 6 hours, and hydrocodone-acetaminophen 10/325 mg daily with only minimal pain relief. Shortly after the AKA despite medication and non-pharmacologic therapies, she reported severe neuropathic pain described as “lightning bolts,” which increased in frequency and intensity. In addition, she reported limitations in mobility, dependence on caregivers for activities of daily living, and significant impact on quality of life.

Despite engagement in rehabilitation and prosthetic fitting, the patient continued to experience debilitating neuropathic pain. She initially wore a prosthetic but reverted to wheelchair use due to pain caused by a sciatic neuroma as well as excess medial thigh skin that impaired prosthetic fitting. Prolonged use of a wheelchair and crutches led to overuse musculoskeletal complaints, including trigger thumb. Despite trials of physical therapy, non-opioid and opioid analgesics, and other conservative measures, her pain persisted.

In early 2025, she was referred to the Rush Pain Clinic for a trial of SCS. Following an SCS trial that resulted in 80% pain reduction after two weeks, a permanent system was implanted, with two epidural leads positioned to span approximately the T8-T10 vertebral levels (Figure 1).

**Figure 1:**
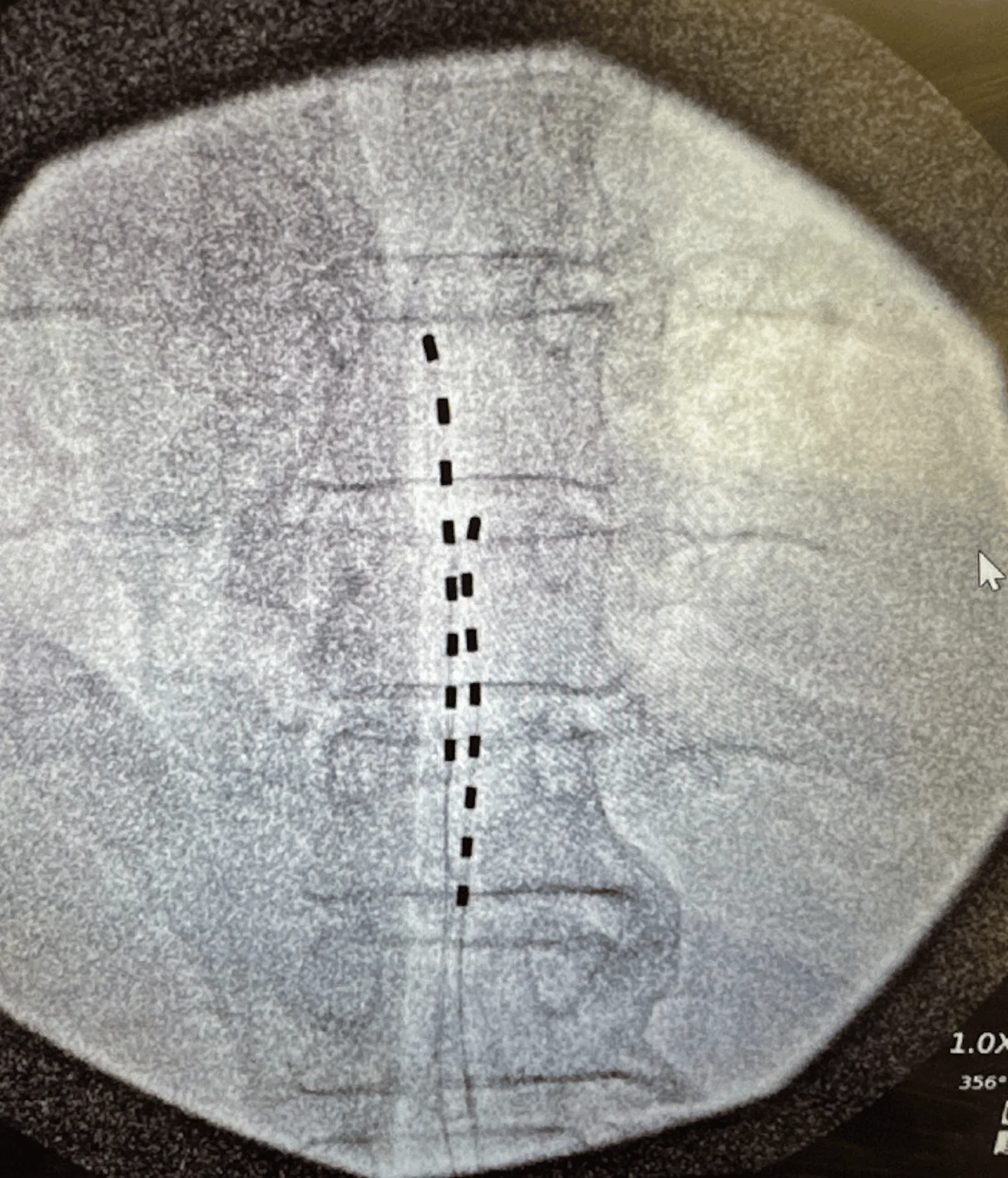
AP fluoroscopic view demonstrating SCS lead placement. Fluoroscopic image in the AP projection showing two percutaneous epidural leads positioned in the midline dorsal epidural space. The electrode contacts are visualized as radiopaque segments spanning approximately the T8–T10 vertebral levels. AP, anteroposterior; SCS, spinal cord stimulation

Two weeks after permanent SCS implantation, the patient underwent excision of the right sciatic neuroma with TMR because persistent neuroma-related pain remained clinically significant despite the initial response to SCS. During the procedure, the tibial component of the sciatic nerve was transferred to a motor branch to the semitendinosus muscle, and the peroneal division of the sciatic nerve was coapted to a motor branch supplying the biceps femoris/lateral hamstring. Because of a significant size mismatch between the nerves, a muscle graft was wrapped around the coaptation [7]. A right medial thighplasty was also performed to remove excess skin and improve prosthetic fit. Figure 2 shows the sciatic nerve with distal neuroma, with white vessel loops wrapped around the motor branches to the medial and lateral hamstrings. The neuroma was excised, with the residual tibial component of the sciatic nerve coapted to the medial hamstring motor branch. The peroneal division of the sciatic nerve was transferred to the designated motor branch of the lateral hamstring/biceps femoris.

**Figure 2:**
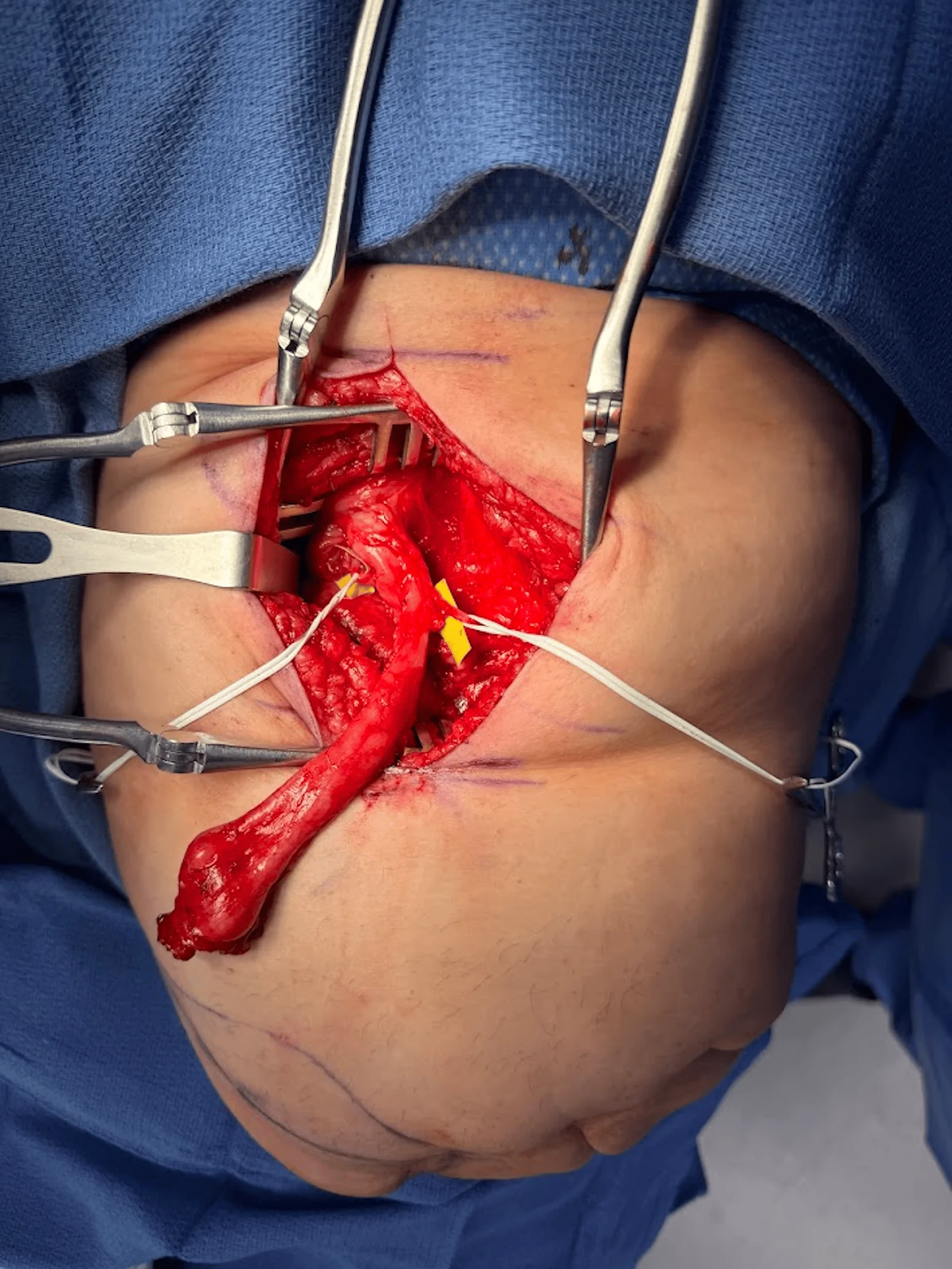
Intraoperative photos during TMR procedure The sciatic nerve with distal neuroma can be visualized, with white vessel loops wrapped around the motor branches to medial and lateral hamstrings. TMR, targeted nerve reinnervation

The patient was assessed using the Numerical Rating Scale (NRS), Patient Global Impression of Change (PGIC), and the PROMIS Sleep Disturbance [8-10]. Prior to the procedure, the patient rated her PLP as a 7 out of 10. Her pain scores decreased to 4-5 shortly after treatment; however, because SCS and TMR were performed sequentially, the relative contribution of each intervention cannot be determined. At the one-month follow-up, the patient reported a pain score of 4 and no longer required oral analgesics (Table 1). This relief in pain remained consistent during her second- and third-month follow-up, demonstrated with NRS scores of 3 and 4. The patient’s global impression of change improved from feeling “moderately better” to “definite improvement,” demonstrating the operation’s success in reducing pain. While we did not see much change regarding the patient’s sleep disturbances, her T-score average of 52.9 still falls within normal limits. This indicates that although her sleep disturbance is slightly worse than the general population, it is not concerning for clinical intervention.

**Table 1:** Pain scores (NRS) collected before and after TMR procedure, as well as PGIC and PROMIS scores *Based on patient recall NRS, Numerical Rating Scale; SCS, spinal cord stimulation; PGIC, Patient Global Impression of Change; TMR, targeted nerve reinnervation

	Two days pre-operatively	At the 1-month follow-up	At the 2-month follow-up	At the 3-month follow-up
NRS (0-10)				
Prior to SCS treatment*	7	9	10	10
Since SCS treatment	4	3	3	4
Current	4	2	2	3
% of pain relief since SCS treatment	30%	80%	90%	80%
PGIC	Moderately better	Considerable improvement	Definite improvement	Definite improvement
PROMIS Sleep Disturbance 8a	53.4	53.4	53.4	51.3

## Discussion

This case describes sustained pain improvement following sequential SCS and TMR in a patient with refractory PLP; however, the relative contribution of each intervention cannot be determined from this single case. The novelty of this report lies in the perioperative use of SCS alongside TMR, drawing attention to its potential as a complementary strategy when conventional pharmacologic treatments fail. Although this is a single case, it illustrates the potential benefits of combining neuromodulation with nerve reinnervation surgery. The patient experienced sustained pain relief of approximately 80% for at least three months after SCS implantation with burst stimulation, despite failing prior pharmacologic management. This highlights SCS as a component in the multimodal treatment of PLP.

Notably, existing literature provides precedents for combining neuromodulation with surgical reinnervation. Agrawal et al. reported significant pain improvement in a post-amputation patient who received a peripheral nerve stimulator placed alongside TMR [11]. Moreover, Raasveld et al. demonstrated a proof-of-concept study on a small number of patients treated with combined peripheral nerve surgery and peripheral nerve stimulation, in which six of seven patients reported meaningful pain relief for over 2.4 years of follow-up [12]. These reports support the use of neuromodulation in addition to surgical nerve reconstruction to provide benefit for patients with amputation pain. Our case further extends this paradigm by using central neuromodulation (SCS) rather than peripheral stimulation, suggesting that more proximal modulation may augment the benefit in complex post-amputation pain.

The exact etiology of PLP is unclear to this day. However, imaging studies including MRI and PET scans demonstrate that brain activity in areas associated with phantom pain of the amputated limb appears to involve both peripheral and central nervous systems [13]. Hence, the rationale for utilizing SCS for the patient was based on a variety of factors, including patient preference, physician preference, and the intended approach to target central nerves to alleviate PLP. Patient-reported pain relief increased from 30% immediately before TMR to 80% at the three-month follow-up (Table 1). However, because SCS remained active throughout follow-up, this temporal improvement cannot establish whether the additional benefit resulted from TMR, continued SCS therapy, or an interaction between both interventions.

Although there are no prior cases that report the use of SCS modulation in combination with a surgical intervention to improve chronic post-amputation pain, there are case reports that demonstrate the benefit of SCS in treating PLP. For example, a recent case report by Sasaki et al. described significant pain reduction and improved quality of life in a patient who underwent SCS implantation 30 years after onset of constant PLP [14]. Another report by Viswanathan et al. conducted a case series involving four patients with intractable PLP who underwent SCS [15]. All patients reported excellent pain relief, with three showing a two-point decrease on the MRS and improvements across multiple symptom dimensions. These findings highlight the potential of SCS to modulate central pain pathways and support its role as an adjunctive therapy alongside peripheral surgical intervention. In line with this, Markewych et al. emphasize that combining neuromodulation with other treatment modalities, including pharmacologic and surgical interventions-such as TMR, RPNI, or dorsal root ganglion stimulation, may offer the most effective outcomes for managing neuropathic neuroma pain, supporting the rationale for a multimodal approach in complex cases like ours [3].

Many patients experience a temporary worsening of RLP and PLP in the initial months following TMR and RPNI [16]. Notably, our patient did not exhibit this post-operative increase in pain. This observation underscores the potential clinical value of combining SCS with TMR, suggesting that a multimodal approach may improve both short- and long-term outcomes in chronic amputation-related pain.

## Conclusions

This case demonstrates the feasibility of sequential SCS and peripheral nerve reconstruction in a patient with refractory post-amputation pain, while providing hypothesis-generating evidence for a potential complementary interaction between central and peripheral treatment strategies. Our patient findings serve as evidence for the concept of SCS being a complementary modality when surgical and pharmacologic treatments are insufficient. Further studies are warranted to explore optimal timing, patient selection, and long-term outcomes for this combined approach.
